# Deterministic modelling of seed dispersal based on observed behaviours of an endemic primate in Brazil

**DOI:** 10.1371/journal.pone.0244220

**Published:** 2020-12-28

**Authors:** Nima Raghunathan, Louis François, Eliana Cazetta, Jean-Luc Pitance, Kristel De Vleeschouwer, Alain Hambuckers

**Affiliations:** 1 UR SPHERES, University of Liege, Liège, Belgium; 2 Graduate Program in Ecology and Biodiversity Conservation, Applied Ecology and Conservation Lab, Universidade Estadual de Santa Cruz, Ilhéus, BA, Brazil; 3 Centre for Research and Conservation, Royal Zoological Society of Antwerp, Antwerpen, Belgium; 4 Bicho do Mato Research Institute, Belo Horizonte, MG, Brazil; Universita degli Studi di Firenze Dipartimento di Biologia, ITALY

## Abstract

Plant species models are among the available tools to predict the future of ecosystems threatened by climate change, habitat loss, and degradation. However, they suffer from low to no inclusion of plant dispersal, which is necessary to predict ecosystem evolution. A variety of seed dispersal models have been conceived for anemochorous and zoochorous plant species, but the coupling between vegetation models and seed dispersal processes remains rare. The main challenge in modelling zoochoric dispersal is simulating animal movements in their complex habitat. Recent developments allow straightforward applications of hidden Markov modelling (HMM) to animal movements, which could ease generalizations when modelling zoochoric seed dispersal. We tested the use of HMM to model seed dispersal by an endangered primate in the Brazilian Atlantic forest, to demonstrate its potential simplicity to simulate seed dispersal processes. We also discuss how to adapt it to other species. We collected information on movement, fruit consumption, deposition, and habitat use of *Leontopithecus chrysomelas*. We analysed daily trajectories using HMM and built a deterministic Model Of Seed Transfer (MOST), which replicated, with good approximation, the primate’s movement and seed deposition patterns as observed in the field. Our results suggest that the dispersal behaviour and short daily-trajectories of *L*. *chrysomelas* restrict the species’ role in large-scale forest regeneration, but contribute to the prevalence of resource tree species locally, and potentially maintaining tree diversity by preventing local extinction. However, it may be possible to accurately simulate dispersal in an area, without necessarily quantifying variables that influence movement, if the movement can be broken down to step-length and turning angles, and parametrised along with the distribution of gut-transit times. For future objectives, coupling MOST with a DVM could be used to test hypotheses on tree species survival in various scenarios, simulating regeneration and growth at regional scales by including data on main dispersal agents over the area of interest, distribution of tree species, and land use data. The principal advantage of the MOST model is its functionality with data available from the literature as the variables are easy to parametrise. We suggest using the coupled model to perform experiments using only available information, but varying the numbers and species of seed dispersers, or modifying land cover or configuration to test for possible thresholds preventing the extinction of selected tree species.

## Introduction

In tropical ecosystems particularly, zoochory is the dominating mechanism by which tree seeds are dispersed. The animals’ movement guided by several environmental factors, coupled with gut transit time, ultimately determines seed deposition pattern [1]. Many studies have shown that the movement of animals (or groups of animals) within their home ranges depends on the spatial distribution of resources like fruiting tree species, or more generally, their food resources, water bodies, or sleeping sites (e.g. [2, 3]). Other factors that could influence trajectories, such as canopy cover, might limit visibility to predators and increase connectivity. The presence of liana conglomerates might offer protection from rain and high temperatures, while proximity to humans, and seasonality or weather conditions may also influence species’ daily paths [4–6]. Also, for territorial species, potential conflict zones with other neighbouring individuals or groups can also influence their daily trajectories [7].

Tree species are sessile organism whose survival depends on seed dispersal processes, face a dual risk from climate change, besides an immediate risk from defaunation (i.e. loss of frugivores), that can restrict their survival. Indeed, we are facing the sixth mass extinction event [8]. This current wave of species extinction could be attributed to a synergy among processes such as habitat loss, ecosystem degradation, and climate change. In areas where moving in altitude remains a possibility, the number of plant species is increasing on the mountain summits of Europe [9], species are at higher risk on their warmer trailing edge, as, for instance, demonstrated with the retreat of the Atlas cedar in Moroccan mountains during the last decades [10]. In lowlands, plant migration largely lags behind climate warming [11]. The ability to foresee how these threats could affect biodiversity in the future is of paramount importance for conservation planning. It can be suggested that in lowland areas, opportunities for seeking appropriate climate are limited.

In theory, Dynamic Vegetation Models (DVMs) could simulate future plant composition and productivity for terrestrial ecosystems under different transitory environmental conditions (see for instance [12, 13]). DVMs could also be coupled with agent-based models predicting land use change [14], thus giving the opportunity to map the evolution in the course of time of suitable areas and their connections. However, vegetation modelling often suffers from low to no inclusion of plant dispersal, while this is necessary to predict the evolution of forest ecosystems [15]. To include plant dispersal in modelling, an approach was developed relying on a cellular automaton that limits pixel occupancy on a suitability map for future time slices, by computing dispersal from already occupied pixels [16]. While very useful to estimate dispersal rates, this method does not permit computing the dispersal rate while taking into account any variations in environmental conditions. Another approach is based on the mathematical description or on the mechanical modelling of the dispersal processes. This was previously done for seeds of wind-dispersed tree species [17–19] animal-dispersed species [20] and also in theory [21].

The main challenge in modelling zoochoric dispersal is simulating animal movements in their complex habitat. This problem is reflected in the variety of approaches implemented in the models described above and their limited scope because of a lack of operating instructions to include several animal species. For example, i) Russo et al. [20] focused on the seeds’ movement rather than on animal movements which imply intensive field studies, ii) Levey et al. [22] conceived a movement sub-model very specific for the conditions they studied (harvested patches in a mature forest), iii) Boyer & López-Corona [21] finely analysed the effect of the distribution of animal plant resources but their methods remained theoretical and the models seem difficult to parametrise, iv) Bialozyt et al. [23] needed a deep knowledge of behaviour of their focal dispersing agent as well as environmental characteristics to build an agent-based model, which may be delicate to parametrise while, v) Nield et al. [24] made an in-depth statistical analysis of the trajectories but without taking into account the influence of habitat heterogeneity. However, recent developments allow a straightforward application of Hidden Markov Modelling (HMM) to animal movements, which could ease the generalization of a model of animal seed dispersal. In HMM, the time series of positioning data are processed to obtain angle and step time series. These new series are used for the identification of changes in state or behaviour (step lengths, and turning angles) and the computation of probabilities of switching between states also taking into account local environmental parameters [25–28].

Our objective was to build a deterministic model of seed dispersal by a primate species that could be further adapted to other animal species. We collected field data to parameterize and validate the projected model. We focused the observations on a group of the endangered species *Leontopithecus chrysomelas* (Golden-headed lion tamarins, hereafter tamarins) in Brazilian Atlantic Forest (BAF) interacting with fruits produced by *Pourouma* spp. trees. We first established the daily trajectory of a habituated group of tamarins, and recorded fruit consumption and seed deposition events, in combination with environmental variables. Then, we analysed the movement within the home range using HMM in relationship with environmental variables. Finally, we conceived and coded, using Fortran 90 programming language [29], a model of seed deposition with random components by combining HMM results, habitat characteristics and gastrointestinal transit time, which could be validated by comparing its outputs with the deposition data from the field. We further examined how to couple with a DVM and adapt to other animal and plant species.

## Methods

### Study sites

The fieldwork took place in 2015–2016 in the district of Colônia, within the municipality of Una, Southern Bahia, Brazil (15° 17’ 8” & 39° 8’ 1”; SISBIO permit number 47178–1). The forest is classified as lowland Atlantic rainforest. The region has an average annual precipitation of ~2000 mm/year, and average annual temperature of 24°C. The study area (hereafter Colônia) is comprised mostly of regenerating areas, that were formerly rubber (*Hevea brasiliensis*), or rubber and cacao (*Theobroma cacao*) plantations. Some areas within the mosaic are still active for shade-grown cacao harvesting or banana production, with some plantations harbouring a combination of rubber with banana. De Vleeschouwer and Oliveira [30] describe the characteristics of the rubber plantations in this area, suggesting potential reasons for the tamarins to frequent such areas, despite anthropogenic activity that occurs within this habitat. The forested area is interspersed with some manioc plantations (*Manihot esculenta*), not used by the tamarins, and therefore not considered as part of their home range. We also used location data from two other tamarin groups, which came from a 2006 study that took place in the Una Biological Reserve. Una Biological Reserve is comparable to Colônia since both areas are situated in the Una municipality, with similar rainfall patterns and a largely proportion of regenerating forests area. At Una Biological Reserve however, there are no anthropogenic activities, and there is a larger proportion of advanced secondary forests, largely due to its protected status and undisturbed natural regeneration [31].

### *Pourouma* trees in the study site

We focus this study on tamarin dispersal of *Pourouma* seeds due to the fact that *Pourouma* seeds are one of the largest that tamarins can swallow (~11.9 mm by 10.5 mm), and also due to the long fruiting season of the species each year (typically from mid/end October to late April). In areas with high levels of defaunation, large-seeded species are at higher risk of local extinction [32]. The level of defaunation was not directly quantified in our study site, but the region is known to be defaunated [33]. There were at least 3 species of *Pourouma* in the Colônia study area, and we observed that the fruiting period began and ended between November and April.

### Primate groups

All applicable institutional and/or national guidelines for the care and handling of animals were followed. Animal handling complied with the protocols approved by the Ethics Committee on Animal Experimentation at the Universidade Estadual de Santa Cruz (number 13/07). At Colônia, we studied one group of tamarins composed of 4 adults (2 males, 2 females) in the beginning of the study, and four adults, one juvenile, and one infant at the end of the study. In Una Biological Reserve the two groups had 6 individuals each (group 1: 4 adults, 1 juvenile, 1 sub-adult; group 2: 3 adults, 1 juvenile, 2 sub-adults). The habituation process [34] was completed prior to the onset of observations for all the three groups and followed the protocol described by Dietz et al. [35]. At least one adult individual in each group had a radio collar affixed, to follow the groups using radio-telemetry. The groups were observed from sleeping site to sleeping site (i.e. from the moment they left the sleeping site in the morning, until the end of the day when they return to the same or a different sleeping site). Every 20 minutes, we noted the coordinates using a Garmin etrex 30 GPS. Only complete observation days were used for analyses (26 days for Colônia group, 46 and 50 for the two Una Biological Reserve groups). In both study sites (Colonia and Una Biological Reserve), groups were followed between 4 to 6 days per month, with at least one day per week of complete observations. Weather was the principal reason for “incomplete” days, i.e. not able to remain in the field until the group reached their sleeping site.

For the Colônia group, behaviours relating to feeding, territoriality, predator presence, were recorded using all-occurrence sampling, while resting/grooming/play, were recorded using scan sampling, though were not used in this analysis. Territoriality behaviours included direct encounters with other groups. Vocalisations linked to territoriality were recorded, but not used in the analyses. Predator presence was recorded both via alarm calls (not all predators were visible), and vigilance behaviours. Any movement away from a position following an alarm call was considered a direct response to predator presence. Additionally, for the Colônia group, faecal samples were collected ad libitum. Locations of all scat deposits with *Pourouma* seeds were marked with GPS, and dispersal distances from parent-trees were estimated, based on average gut-transit time. The estimated average gut retention times in *Leontopithecus rosalia* was 74 minutes (+/- 18 minutes) [36]. In this field study, the time difference between the deposition of the faecal sample containing a seed of interest, and the feeding behaviour observations permitted an average calculation of seed transit time of 75.20 minutes (standard deviation = 19.50; S2 Fig), which was concordant with *L*. *rosalia*. We estimated passage through digestive tract calculating the interval between defecation containing seeds (faecal samples without seeds were not included in the analyses) and the prior feeding behaviour of the species’ in the sample by analysing the combination of seeds found in the faecal sample. When the most likely parent tree was corroborated (in some instances, a mean distance was calculated if the group consumed fruits from individuals of the same species within minutes and metres of each other), the defecation distance between the faecal sample and parent tree was calculated, using ArcMAP v. 10.5.1. The seasonal home range area was also calculated using the minimum convex polygon in ArcMap10.5.1., though we also identified the 95% kernel (P95%)–i.e. the zones where the probability of encountering the group is 0.95. For the purposes of this study, the home range was calculated during one fruiting season of *Pourouma*. This should be considered a seasonal home range, as the full extent of the group’s home range was not evaluated due to the limited field time. For the two groups from Una, complete days covered a whole year, for this reason the observation days was almost two times higher.

### Environmental data from Colônia de Una (Colônia)

To test for possible environmental variables that could influence tamarin movement and validate the model, we set up 14 transects spaced 50 m from each other. In each transect we installed 15x15 m plots each 15 m apart. The number of plots within each transect varied based on transect length that range from 2 to 9, and we ended with a total of 70 (15m x 15m) plots in the home-range corresponding to the fruiting season. The number of plots per transect varied based on the length of the transect, which ran south-north between the southernmost and northernmost limits of the home-range corresponding to the fruiting season of Pourouma species. The distance between 15m x 15m plots was 50m, and the distance between one plot and the next along the same transect (i.e. from the northernmost side of one plot and the southernmost side of the next) was 15m. In each plot we evaluated the basal area of fruit resources, and the leaf area index. We did not distinguish between male and females of dioecious species. Individuals of species known as fruiting resources were considered as potential resources and the basal area was calculated. In each plot, the trees were identified (at least to family level) for all individuals with a DBH > 5cm and the basal area of the known fruit trees was calculated based on the DBH. To estimate leaf area index, we took hemispherical pictures of the canopy. The pictures were taken using a Sigma 8mm f/4 circular Fisheye lens (Equi-angular projection) following the methods described in Bequet et al. (2012) [37] at 9 points in each plot; the pictures were analysed with Hemisfer v.2.16 software [38, 39].

Additionally, over a one-week period, during the fruiting peak of the species, we identified and mapped more than 600 *Pourouma* individuals and calculated the Fournier score [40] to obtain a snapshot of *Pourouma* fruit availability during the season. To obtain a Fournier score, the percentage of fruits (ripe and unripe) available was scored on a scale of 0 (no fruit) and 1 to 4 (25% to 100%). Less than 25% of the *Pourouma* individuals have mature fruit at any given time during the fruiting season. The fruit availability index was calculated by multiplying the Fournier score for presence of ripe or unripe fruit with the diameter at breast height (DBH) of all *Pourouma* fruit trees that the tamarins consumed. Finally, we interpolated fruit availability index (FAI), basal area (BA), and leaf area index (LAI) over the entire seasonal home range, using ordinary kriging in ArcMAP to obtain continuous maps. Note that for the two primate groups from Una Biological Reserve, comparable environmental data was not available. The behavioural data from Una Biological Reserve were collected at an earlier date, as part of a separate research project. The behavioural data collection methods were identical, and therefore comparable between Colônia and Una Biological Reserve groups, but data on other biotic and abiotic characteristics were not available for Una Biological Reserve to allow similar analysis at the time of this research.

### Golden-headed lion tamarin movements

Markov modelling requires positioning data at regular time slices. For each primate group, we prepared a single time series, by joining the successive coordinates of all the complete observation days end-to-end. We used the gamma distribution for the step lengths and the von Mises' distribution for the angles, based on the goodness of fit. We determined the number of states, by comparing the Akaike information criterion (AIC) of the possible models. Various “physical” environmental variables (FAI, BA and LAI from the kriging maps) were tested as covariates, for increased model fitness. Other factors, more linked to group behaviour, include distance from the GPS location to: sleeping site, fruiting trees, bromeliads, predators, other groups (i.e. encounter behaviours), and resting sites. Not all possible model combinations were evaluated. After comparing the AIC values for individual covariates against the null hypothesis, we selected those covariates with the lowest values and tested fitness of combinations. We used distances to these sites as a proxy for “attractors” and movement towards the “closest attractor”, exceptions being predators and encounters. We used the moveHMM v1.2 R package [41] to perform the analyses. The transitions between states were also computed by the package with the equations provided in [41].

### Deterministic Model of Seed Transfer (MOST)

**Fig 1:**
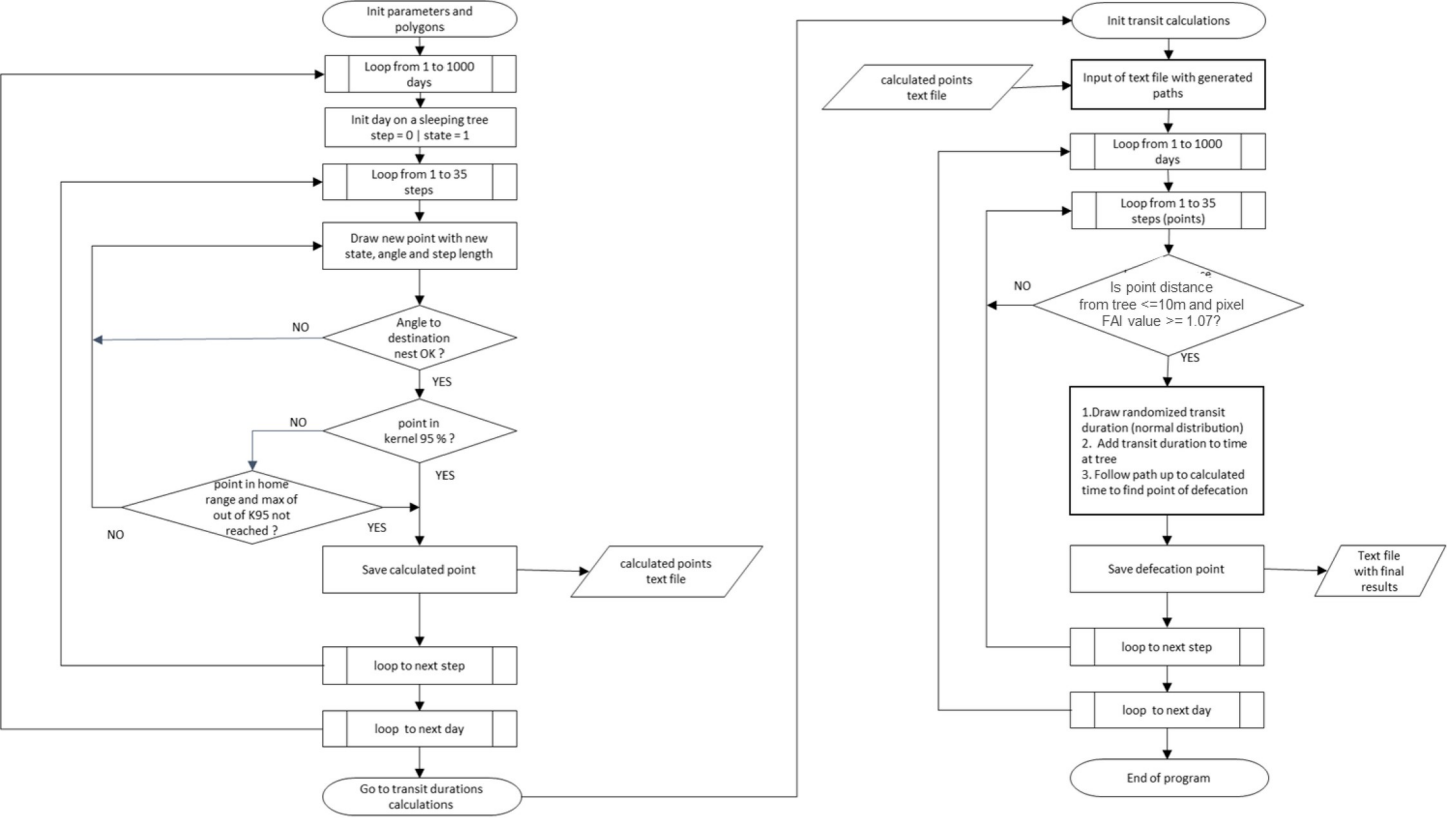
Deterministic Model of Seed Transfer (MOST) algorithm created in Fortran 90 programming language, using parameters extracted from HMM’s two state model.

The model (MOST for Model Of Seed Transfer) was written in Fortran 90 [29] to allow spatial extensions and further coupling with a DVM (CARbon Assimiliation In the Biosphere, CARAIB) also written in this language [13]. Most of the constants describing the home range, the movements and the animal behaviour, may be changed in a parameter file before executing MOST. We considered plantations and agricultural zones as “no-go” zones (non-habitat areas), in this iteration of MOST, to simplify the real-world observations. This was also due to the fact that no *Pourouma* trees are kept in agro-forestry systems, so no seed swallowing or defecation took place in these areas. In the case of Colônia group, the main sleeping site was also situated at the edge of a plantation area, and the trajectories reflect the short paths that cross a small part of the no-go zone. The model (Fig 1) starts by reading coordinates of the home range and kernel 95% as well as sleeping site positions. A day path counts a succession of 35 steps (successive positions separated by 20 minutes), corresponding to an average day-length of 700 minutes, outside sleeping sites. The initial (morning) and the next selected (evening) sleeping sites were drawn from the re-use frequency. The simulation is initiated by setting the initial state of the HMM to one (note that we selected a two-state HMM) to know which of the distributions to use for angle and step. Accordingly, the first step and the first angle are drawn to reach the first position from the starting sleeping site, using the gamma (step) and the Von Mise's (angle) distributions. Then, MOST verifies where the position is located within the 95% kernel using the "Winding Number" algorithm (Sunday 2001: http://geomalgorithms.com/a03-_inclusion.html#wn_PnPoly()). If the position is not within the kernel but still in the home range and maximum count out of the 95% kernel is not reached (see below) then position is validated. Otherwise, new angles and steps are redrawn until the conditions are satisfied. The characteristics of the new position are recorded and based on the new state the next angle and step to reach the next position. To simulate the end of the day, i.e. arbitrary after 26 steps, on average corresponding in the field to the moment where the animals seemed to head to the sleeping-site, the drawn angles are progressively restricted from 360° to 22.5° to reach the selected sleeping site while the last step automatically drives the group to the selected sleeping site position; possibly, they can arrive sooner when their position are less than 10 m. To be able to match the generated position frequencies within the P95%, we first analysed the density distribution of the number of positions of the observed trajectories outside the P95% using the fitdistrplus R package [42]. The obtained distribution with its parameters was used to generate a table of probable numbers of positions outside P95%. MOST records the trajectories, and stores for each of them its number of positions outside P95% and finally keeps only a subsample of trajectories providing the same numbers of positions outside P95% as table generated from the observed kernel.

*Pourouma* seed swallowing was simulated if the position was within a distance of 10 m of a *Pourouma* density pixel larger than 1, and if the pixel value of *Pourouma* mature fruit score was above 0.30. This information is read from the kriging maps. The distance has to be introduced because the likelihood that a given generated position will coincide directly with a fruit tree is very low. The three thresholds were selected after tuning. We examined the normality of the observed gut-transit time (Shapiro-Wilk normality test; S2 Fig). Seed gut-transit time is simulated by drawing random values with corresponding distribution in the 95% confidence interval of the mean of the observed values, which allows the computation of the coordinates of their deposition further on the trajectory. The simulated spatial dispersal kernels could finally be calculated by recording the distances between the fruit trees and the defecation events.

For the validation of MOST’s results, chi-squared tests for frequency were calculated, to evaluate the model’s success at reproducing the observed distances of seeds in faeces from parent trees, as well as the distances to the closest congeneric.

## Results

### Colonia study site, group behaviour, and seasonal home range

**Fig 2:**
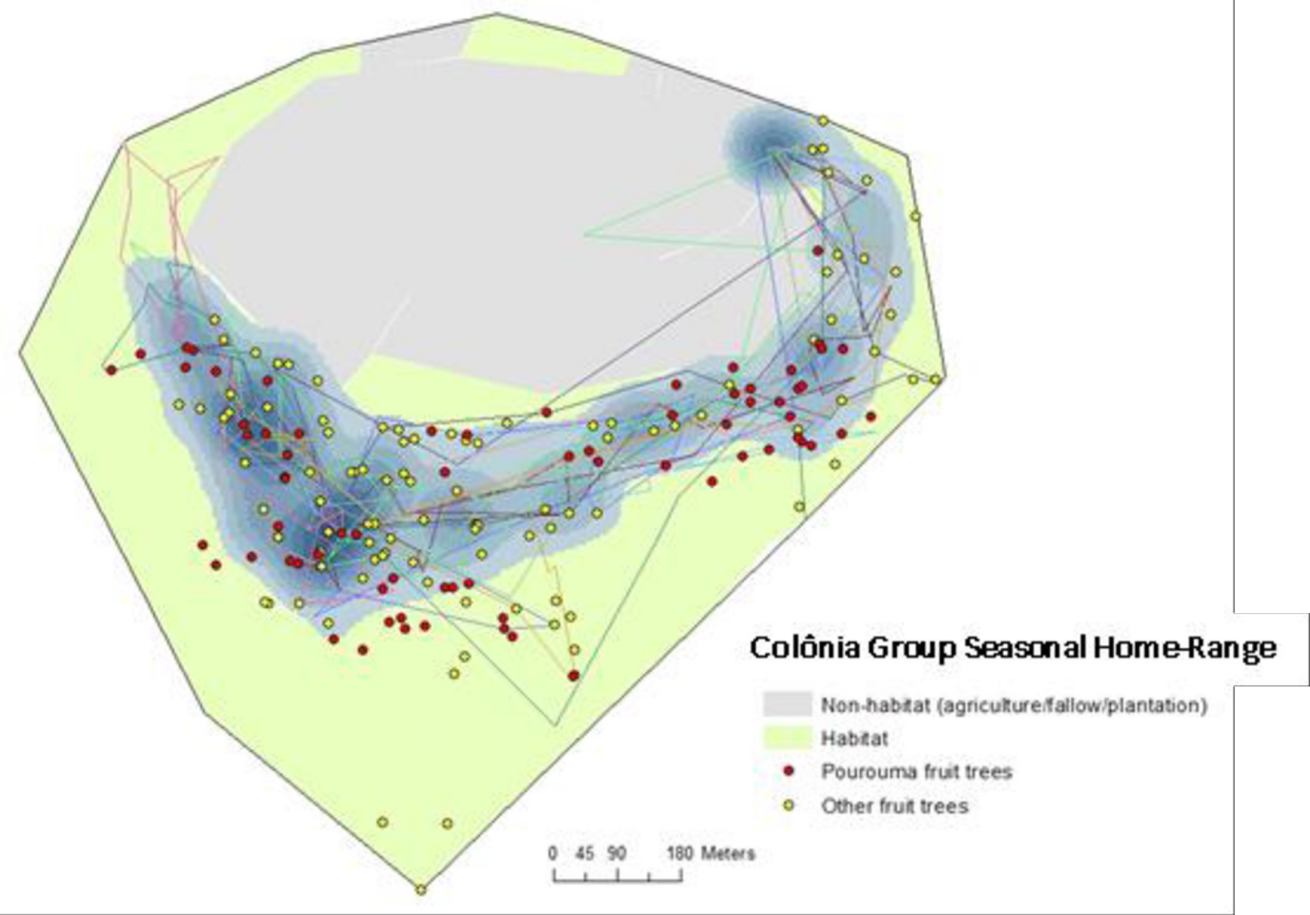
Home range of *Leontopithecus chrysomelas* from Colônia group, Colônia de Una, Brazil during *Pourouma* genus fruiting season (November to April). Coloured lines show some examples of daily trajectories of group (complete days) used for HMM analyses, blue areas show the 95% kernel within the seasonal home range.

The home range corresponding to the fruiting season of *Pourouma* genus trees for the Colônia group was approximately 18.77 ha, composed mostly of secondary forest in various stages of succession (Fig 2). Six sleeping sites were observed in use during this season, with one sleeping site used more than 75% of the times during the study, i.e. the group returned to the same sleeping site as they left in the morning 75% of the times. The seasonal average of the daily path length for the group was 1,867 m, while the recorded positions outside the P95% could be considered as following a zero-inflated Poisson distribution (S3 Fig). During the *Pourouma* fruiting period, over the 26 complete observation days, we observed the group consuming fruits from 148 fruiting trees and flowers from 21 non-tree (bromeliads or lianas) individuals, from at least 30 identified species. Only four species had more than five visits (*Inga affinis*, *I*. *thibaudiana*, *Pourouma* spp, as well as *Artocarpus heterophyllus*), with one *Pourouma* fruit tree having 26 visits during the observation period. Of the 168 foraged individuals (147 fruiting trees, 21 non-tree individuals), 75 were visited only once during the observation period. 79 individual fruit trees belonged to the *Pourouma* species, of which 7 individuals had more than 10 visits during the fruiting season, 17 other *Pourouma* individuals had between 3–10 visits, and the remaining 55 individuals were visited once during the fruiting season (S1 Table).

In the seventy plots, we recorded 2,216 trees, from 203 species, though 103 individual trees were identified only to family level and were excluded as potential fruiting sources. 50% of the species identified are consumed by tamarins, and the *Pourouma* genus represented 3% of the sampled individuals. *Pourouma* mature fruit FAI varied between 0 and 1.99, BA between 18.43 and 40.85 m^2^/ha, and LAI ranged between 0.088 and 2.005 (see S4, S5 and S6 Figs for kriging maps of LAI, BA, and *Pourouma* FAI).

**Fig 3:**
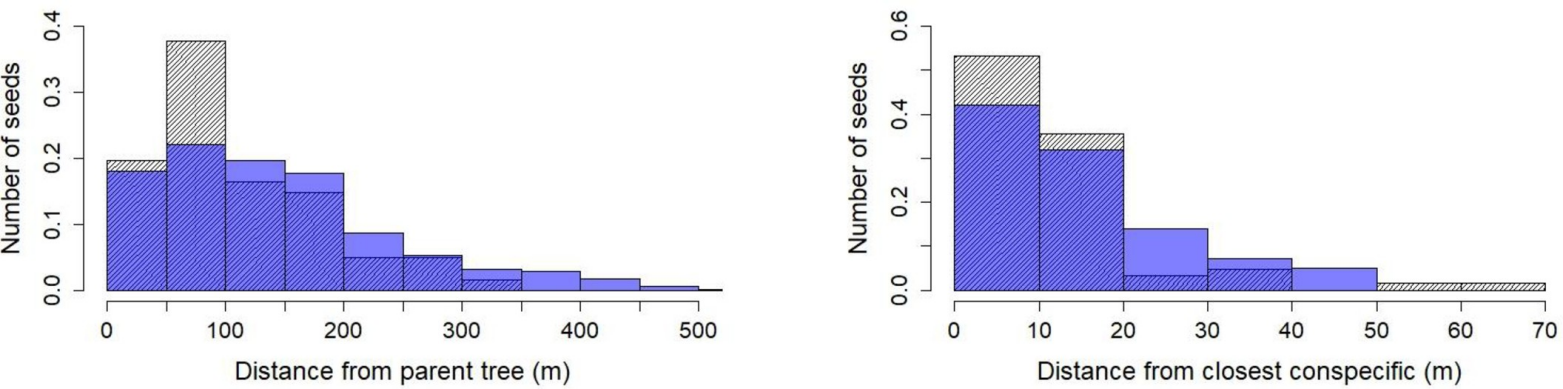
Observed (shaded) and simulated (blue) defecated seed distance densities from parent tree of *Leontopithecus chrysomelas* from Colônia group, Colônia de Una, Brazil (chi-squared = 11.738, df = 6, p-value = 0.06807; left) and from closest *Pourouma* congeneric trees (chi-squared = 78.1645, df = 4, p-value = 0.08573; right).

Average gut-transit times follows a normal distribution (S2 Fig). During the *Pourouma* fruiting season, the Colônia group visited *Pourouma* trees daily. Average dispersal distances of *Pourouma* seeds was 111 m, with peak number of seeds deposited between 60 and 90 m (Fig 3). Given the average transit time in the digestive tract and habitat use, most of the seeds were defecated near congenerics. 80% of all observed defecation events containing *Pourouma* seeds took place within 30m of a congeneric, 20% within 5m of a congeneric tree (Fig 3).

### Model of Seed Transfer (MOST)

For the Colônia group, we selected a two-state HMM, with basal area of fruiting species, along with distance from sleeping sites being key predictors of transition (S2 and S3 Tables), based on the AIC. Parameters used to change from state 1 to state 2 are also provided (transition probabilities). State 1 comprised the longest step lengths, shorter turning angles, and fewer pauses, while state two was characterised by shorter step lengths, larger angles, and more pauses. Testing the model with other co-variates demonstrated higher AIC values and all model combinations’ AIC values were still higher than that of basal area and distance to fruiting trees (S2 and S3 Tables).

**Fig 4:**
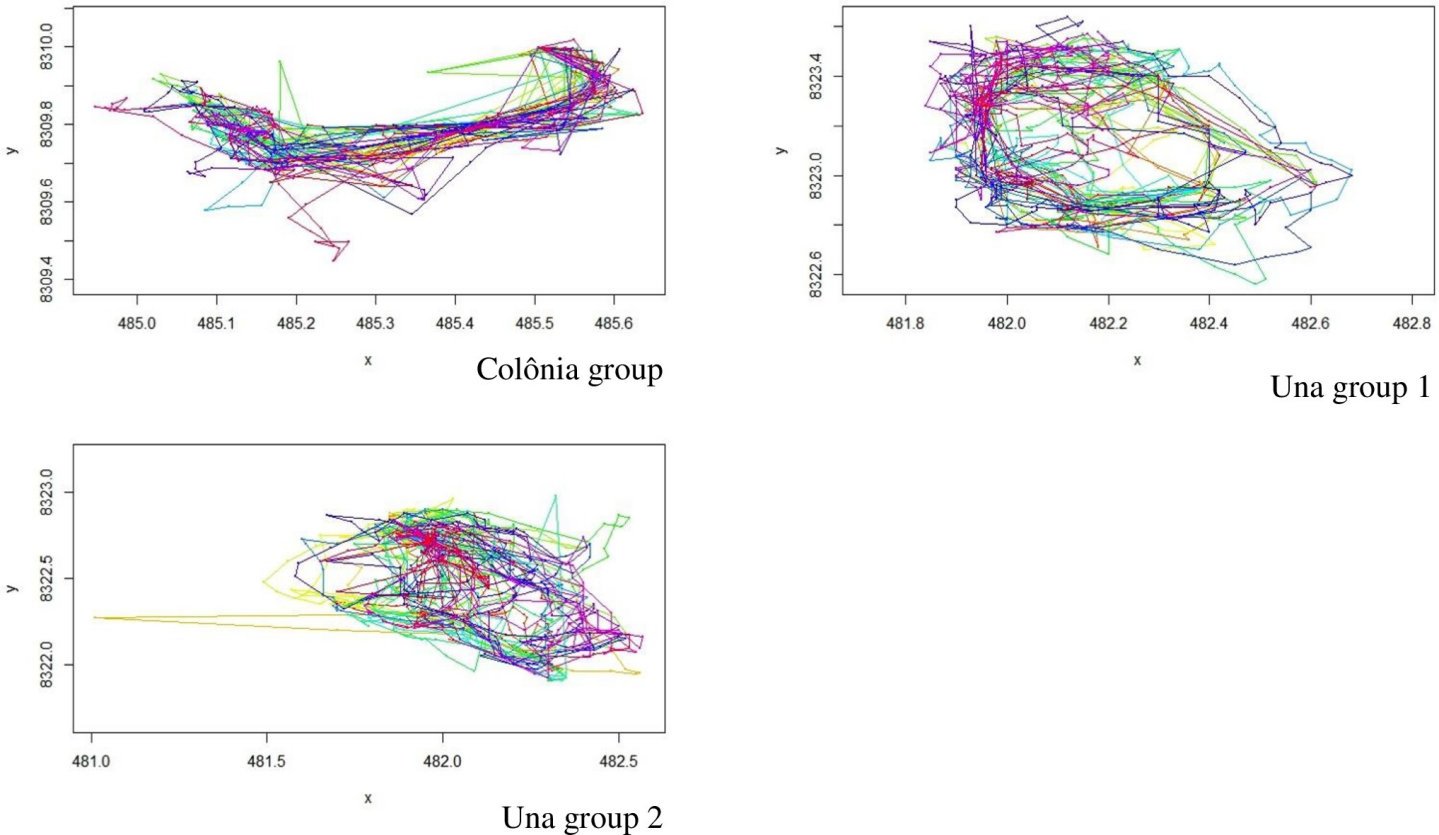
Trajectories of 3 groups (Colônia seasonal trajectories, Colônia de Una, 26 days; Una biological reserve group 1 –annual trajectories, 46 days; group 2 –annual trajectories, 50 days, Brazil), UTM coordinates (UTM Zone 24) on axis. Each colour represents one daily trajectory (of complete days, sleeping-site to sleeping-site).

**Fig 5:**
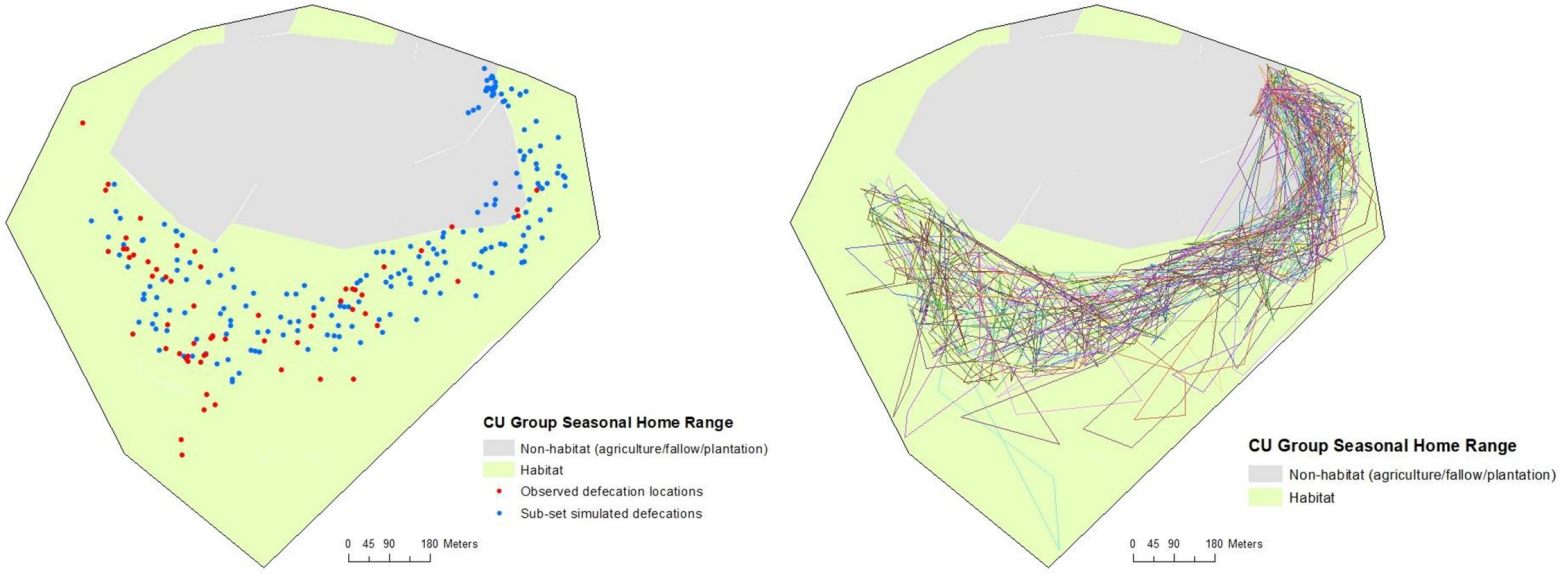
Map of sub-set of simulated defecation events, with distance to *Pourouma* genus tree of 10 m and mature fruit threshold of 0.3, from MOST and observed defecation events within the seasonal home range of tamarins (left) and sub-set of simulated trajectories (right), in Colônia de Una, Brazil.

MOST correctly reproduced the trajectories within the Colônia group’s seasonal home range (Fig 4) but the simulation of seed deposition was sensitive to the threshold value for mature fruit and the distance to *Pourouma* to provoke swallowing, and the final values were obtained through fine-tuning (distance of 10 m and *Pourouma* FAI of 0.3 (Fig 5), see also S7 Fig). According to the chi-squared tests, the simulated distances of seed deposition were not significantly different from those observed (Figs 3, 4 and 5) but the test p-values were close to the thresholds. Distances from parent tree showed a longer tail as expected but it did not appear in distance from closest congeneric.

## Discussion

The deterministic Model Of Seed Transfer (MOST) replicated, with good approximation, tamarins’ movement and seed deposition patterns, as observed in the field. Relative to other seed dispersal models, this method is simpler and can be used to simulate seed dispersal, relying mainly on data obtained from literature, to parametrise or calibrate the model. Such data includes, position data obtained at regular intervals, distribution of key feeding resources, gut transit times. Any additional data on environmental characteristics may be included, such as “go” and “no-go” zones, as we used here. In a more complex home range, or when longer-term data are available, nuances in defining habitat or non-habitat may be relevant. For example, De Vleeschouwer & Oliveira [30] demonstrated that monkeys do in fact use *Hevea* plantations, only when fruit resources and/or bromeliads are available. A frequency table of presence in different habitats can be calculated with more sampling years, to be incorporated into MOST.

The daily trajectories of the tamarin group, and the local factors influencing them are concordant with the literature on variables influencing animal movements: food resource and sleeping site distribution [e.g. 39, 40, 43]. Parameters used to initialise HMM analyses therefore are influenced by habitat composition and home range size. As the choice between the states (2 or more) relies on thorough biological knowledge of the group or individuals of interest, the parameters appropriate for one group may not be relevant for others, as evidenced by the differences between the Colônia and Una Biological Reserve groups (Fig 4), or in another study on macaques in Thailand [44]. The HMM two-state model parameters are directly linked to the home range characteristics of the groups and of course of the species, therefore for each application of the MOST model, environmental characteristics of the home range must be supplied, and this is consistent notably with the theoretical analysis of Boyer & López-Corona [21] who found that the number of movement states should depend on home range heterogeneity. Once the step-length and turning angle are characterised, with as specific as data as possible, updating MOST with parameters is a simpler process than other simulation models, which are too theoretical, agent-based, or lack habitat heterogeneity [21, 23, 24].

The observed dispersal distances are consistent with other studies on *Leontopithecus* genus and *Saguinus* species [45]. However, it is important to note that in the case of generalist frugivores, smaller home range areas with a uniform distribution of fruit resources might dilute any potential patterns in behaviour, particularly linked to feeding. More importantly, our results suggest that within a degraded, mosaic habitat, tamarins may maintain their habitat at a local level or eventually improve the availability of its resource species, but mostly inside their P95% seasonal home ranges. This should not be disregarded because it is probably a mechanism fostering plant species local abundance and preventing local extinction. We hypothesise, that in this specific context of degraded and defaunated areas, species like tamarins had a disproportionate importance. Of course, complementary studies from other frugivores will be very important to ascertain the contribution of tamarins. As they can swallow only up to a certain seed-size, they can help disperse their main fruit resources and maintain tree regeneration within a small area. However, the movement and dispersal patterns of tamarins suggest that they disperse seeds within the habitats where germination and growth is viable, and can therefore at least maintain resource availability. This potential maintenance of the habitat by tamarins is simply that–it does not imply that natural regeneration processes via endozoochory could restore the full level of plant diversity. While it is suggested that zoochoric maximal dispersal distance could be related to home range size [46, 47], this could give a false image of the real dispersal efficiency since it does not take into account seed dispersal away from congenerics.

MOST was able to simulate trajectories resembling the observed data. However, due to the statistical way the HMM is computed, it had to verify that each new step remained inside the home range or in the 95% kernel and if not, redraw values. The moveHMM package uses successions of distances and turning angles for its simulations making it difficult for the simulated movements to mimic real-world constraints that keep animals in a more confined range (R. Langrock, personal communication). However, the package is easy to apply provided the coordinates of the animals are regularly spaced in time and the effort is high enough. We evaluated it as a convenient way to model the trajectories. The seed distribution patterns simulated by MOST were less similar to the observed data. Since more events were simulated rather than observed, we expected to get more extreme dispersal distance values. However, this only happened with distance from parent trees and not with distance from congenerics. This could be caused by the fact that MOST does not use real positions of *Pourouma* and their fruit availability index but kriging maps, which are only extrapolations of real conditions. Moreover, we found that the simulated dispersal distances was affected by the thresholds set for distance from fruiting tree, and fruit availability index which trigger seed swallowing. It may be possible to improve the fit, if necessary, as the user may have an option to simulate scenarios of higher or lower densities of fruiting trees of interest, which can be applied to *in-situ* conservation measures.

Since it could be locally important to compute the seed rain, the modelling of this important ecosystem characteristic has to be included in vegetation models. In order to make the connection between the DVM and MOST and thus simulate the growth of the dispersed seeds, it is first cardinal to take into account the scale of the processes. On the one hand, MOST simulates processes occurring between individuals (trees and animals) on area of some sq. km (here, the home range size was only 0.18 sq. km) while the tree species range can cover areas of thousands of sq. km. Also, DVMs generally consider the growth of the species over domains of this size (i.e. several thousands of square kilometres) returning mean value for whole pixel, not for individuals. Thus, an interface between the DVM and MOST should record tree individual growth from the pixel prediction of the DVM.

The second point to take into account is that seed dispersal generally happens through a network of many animal species. MOST has to be applied for each animal species of interest, for the coupling to reflect the whole dispersal processes of the species of interest. Long-range dispersal is obviously fulfilled by birds, bats and large mammals while the smaller species like tamarins maintain or (possibly) improve tree patches more locally. In our field site, we cannot confirm any potential effect of intraspecific, or intrageneric competition. *Pourouma* saplings (> 10 cm height) were found within short distances of each other, though only in some forest types. In Stevenson (2007), while they found distance-effects on seedlings of *P*. *bicolor*, the evidence for intraspecific competition was not conclusive [48]. In this study, we indeed routinely observed birds and bats during field work as well as other species like *Callithrix kuhlii* (Wied’s marmoset), or *Potos flavus* (kinkajou) reported in the eastern Atlantic Forest [49], feeding on the *Pourouma*. Bats are considered good dispersers because they can travel several kilometres each day, and defecate when flying [50] but it is worthy to note that they generally show high fidelity to swarming sites [51–53] which should produce highly directional dispersal kernel. Seed dispersal by species identified as legitimate dispersers should occur also mainly in their P95% and may also contribute to the clumping of the animal dispersed species [54].

The third point is that it seems nearly impossible to simulate real situations because this requires general, and comprehensive knowledge on density and the home range of all the animal species involved in the processes of dispersion and the distribution of every specimen of the focal tree species over a given area. This exercise would require considerable information and extensive field-work. Some information is available from literature, such as variations in species’ home range size or the gut transit times, and technology progresses will help. Landscape structure is already largely available from remote sensing with increasing precision, up to the possibility to identify tree species, through the use of small drones [55]. Also, animal presence using camera-traps and tracking with GPS systems becomes easier and cheaper [56–59], to compute the HMM characteristics.

The seed dispersal process is affected by habitat loss and fragmentation, and ultimately climate change. As habitats shrink, this causes disturbances in frugivore communities, which carry over on plant communities, due to reduced dispersal. For example, Babweteera and Brown [60] demonstrated that the loss of larger-bodied frugivores decreased the assemblages of larger-seeded, climax tree species in disturbed forest fragments. This was due in part to the specific dependency that larger seeded species had on the presence of large-bodied frugivores, and because the small- & medium-bodied frugivores dispersed the seeds at smaller distances. The scale of fragmentation can affect how individuals or groups interact with distances to find habitat, and therefore alter their typical behavioural states [61]. Animal species that may be highly territorial, or have emigrating individuals, that end up with a large density in a small patch of habitat may suffer from local extinctions if they are unable to broaden their home range [62]. Additionally, in fragmented or degraded landscapes that impact the animal movement, due to varying degrees of permeability within a mosaic, the seed dispersal services can be strongly altered both by loss of vegetation producing fruits [63] or change in fruits characteristics [64] but also due to behavioural constraints of frugivores [54]. Finally, climate change might alter availability of plant resources for the frugivores in the future [13] and it might eventually directly challenge animal survival [65]. Such complex factors may be slightly harder to capture when coupling MOST with a DVM, though various scenarios can be simulated with more or less fragmented landscapes, and more or fewer dispersal agents. Simulating scenarios with fewer or more dispersal agents can help gain a better understanding of the possible impacts of defaunation on habitat regeneration. Similarly, severely fragmented habitats can limit dispersal opportunities, and further degrade habitats. Simulating dispersal movements using MOST could serve as an opportunity to identify potential zones for connectivity and assisted regeneration when chances of relying on natural dispersal mechanisms are limited due to landscape characteristics.

## Conclusion

Movement pattern of our focal species in relation to local habitat characteristics were easily captured using HMM. The information can be transferred in a relatively simple simulator of seed deposition MOST written in Fortran 90. Since simulations with MOST were able to generate quite realistic seed deposition patterns, we concluded that it is valid. Combined with a DVM, MOST can be a useful tool to test hypotheses of tree species survival under climate change by simulating tree regeneration and growth at regional scale. This requires the inclusion of behavioural (trajectories), physiological (gut retention times) and abundance data for the main dispersal agents (spectrum of home range sizes with potential animal densities) over the area of interest, the distribution of the tree (abundance) and land use. When resource constraints preclude field data collection to test on the ground, a feasible approach would be to use the model combination as described above, to perform experiments using already available information, and varying the numbers and the types of dispersers, as well as land and tree configuration to test the existence of thresholds preventing the extinction of selected tree species. Bibliographic literature on species movements, habitat preferences, dispersal behaviour is often more readily accessible and can be valuable inputs to adapting the MOST algorithm.

## Supporting information

S1 Fig (TIF)

S2 Fig (TIF)

S3 Fig (TIF)

S4 Fig (TIF)

S5 Fig (TIF)

S6 Fig (TIF)

S7 Fig (TIF)

S1 Table Frequency of visits of feeding trees in Colônia de Una, during *Pourouma* fruiting season (November–April). (DOC)

S2 Table Selection of the hidden Markov model (HMM) simulating animal movements within the home range of *Leontopithecus chrysomelas*. (DOC)

S3 Table Selected model: BA+SS (basal area of fruiting trees + distance to sleeping sites). (DOC)
