# Supplementary figures and images for: Deterministic modelling of seed dispersal based on observed behaviours of an endemic primate in Brazil
Source: PLoS One. 2020 Dec 28;15(12):e0244220. doi: 10.1371/journal.pone.0244220 (PMC7769435; doi:10.1371/journal.pone.0244220)

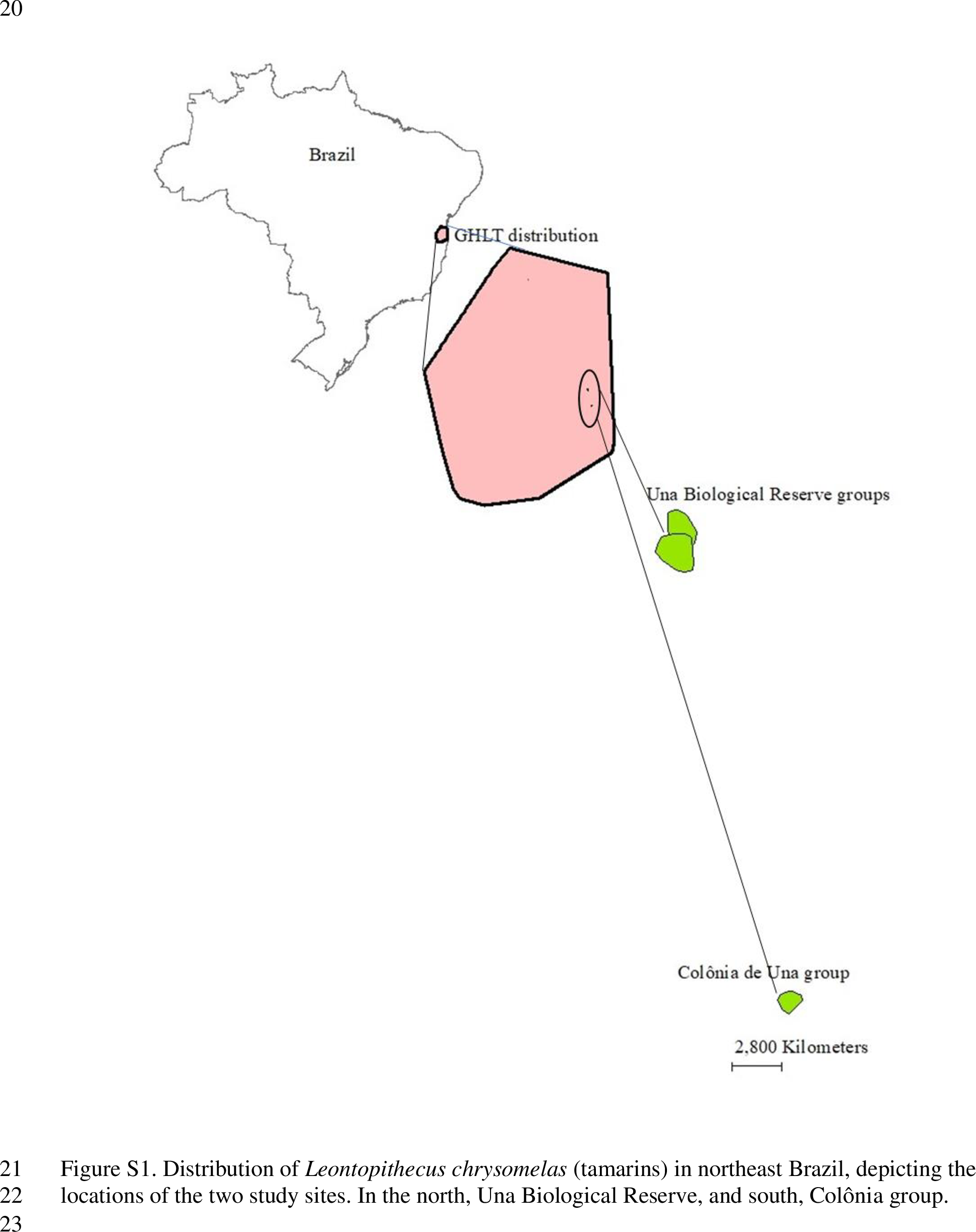

Supplement: S1 Fig — (TIF) [file pone.0244220.s001.tif]

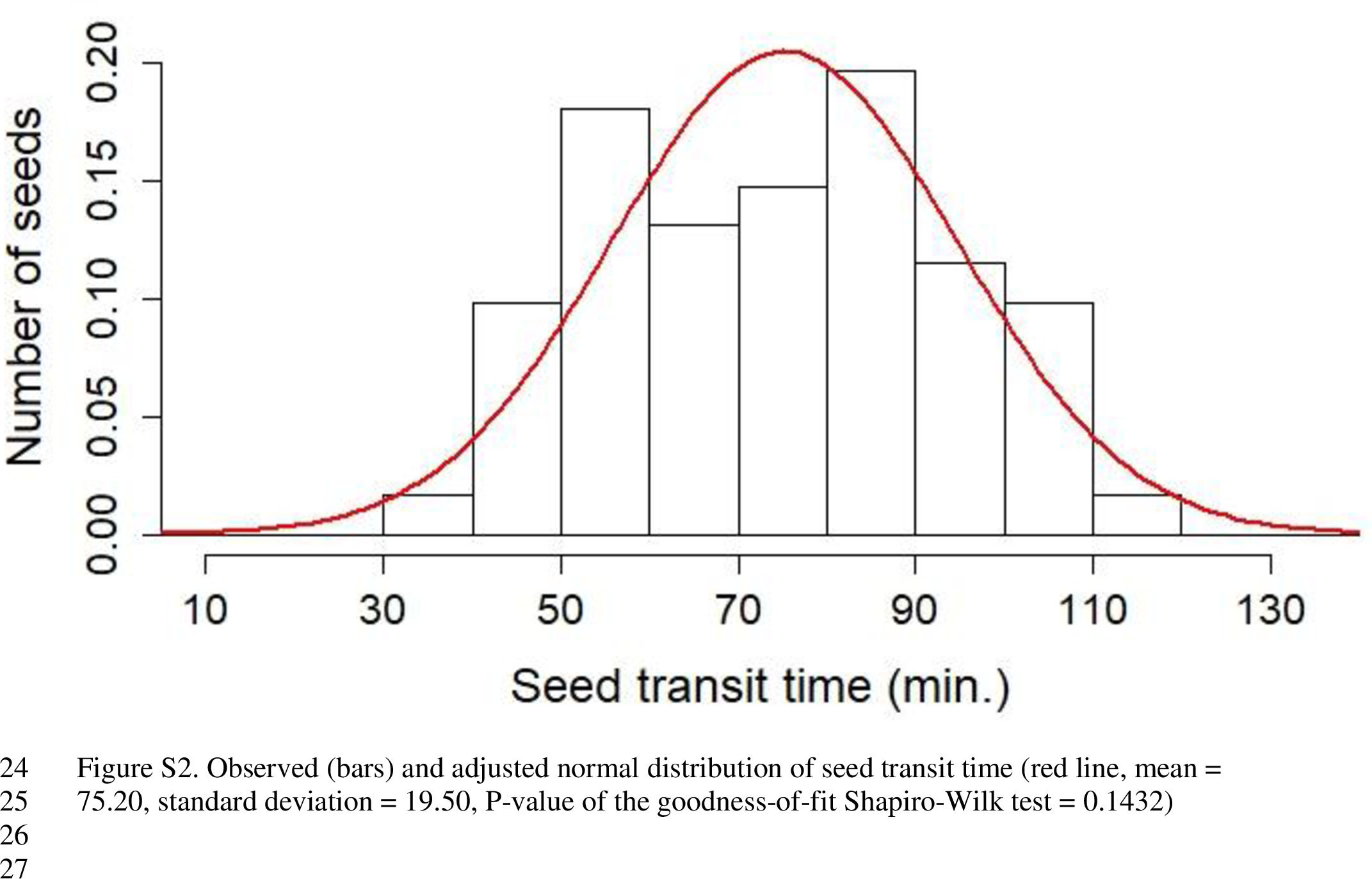

Supplement: S2 Fig — (TIF) [file pone.0244220.s002.tif]

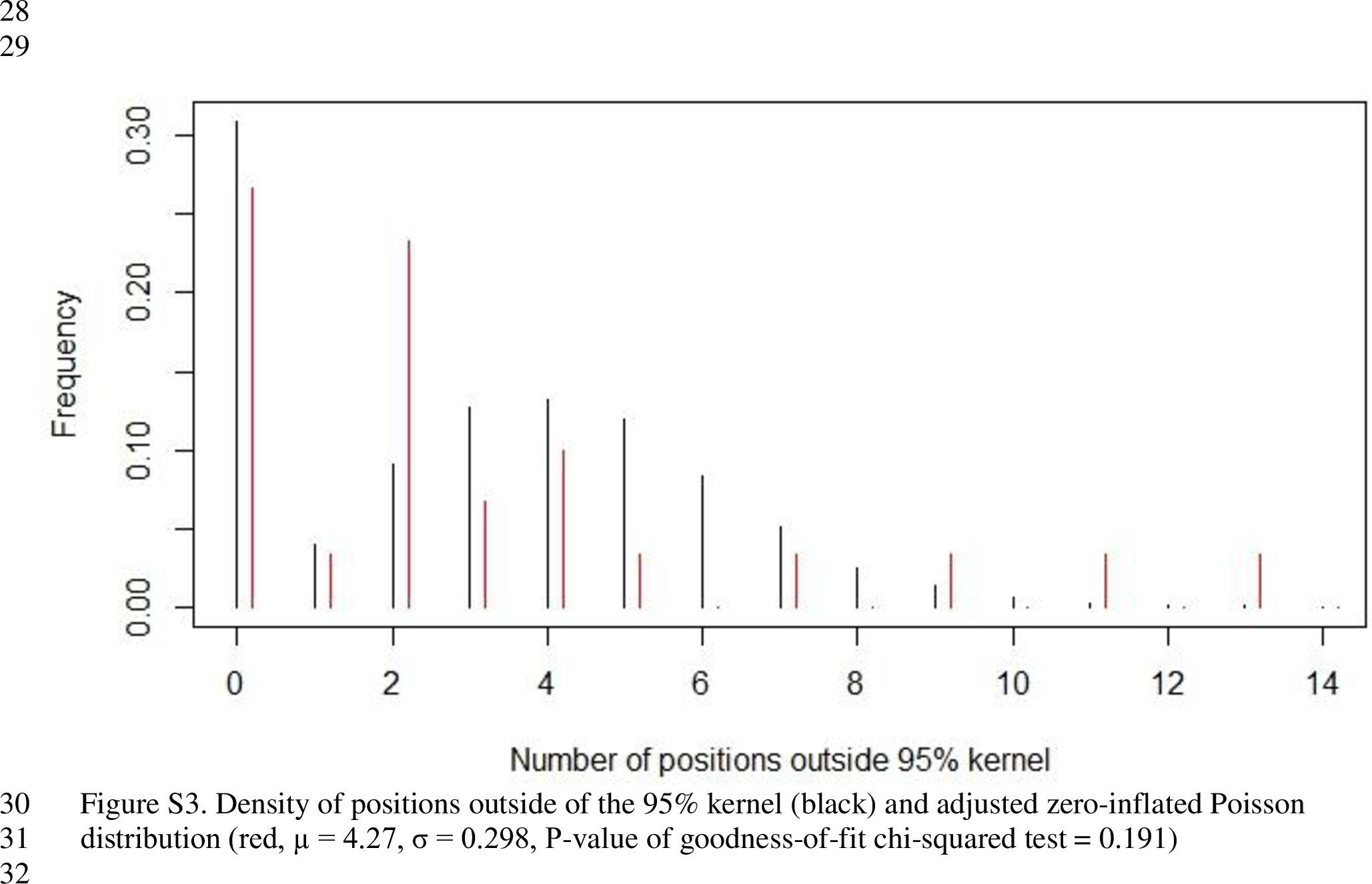

Supplement: S3 Fig — (TIF) [file pone.0244220.s003.tif]

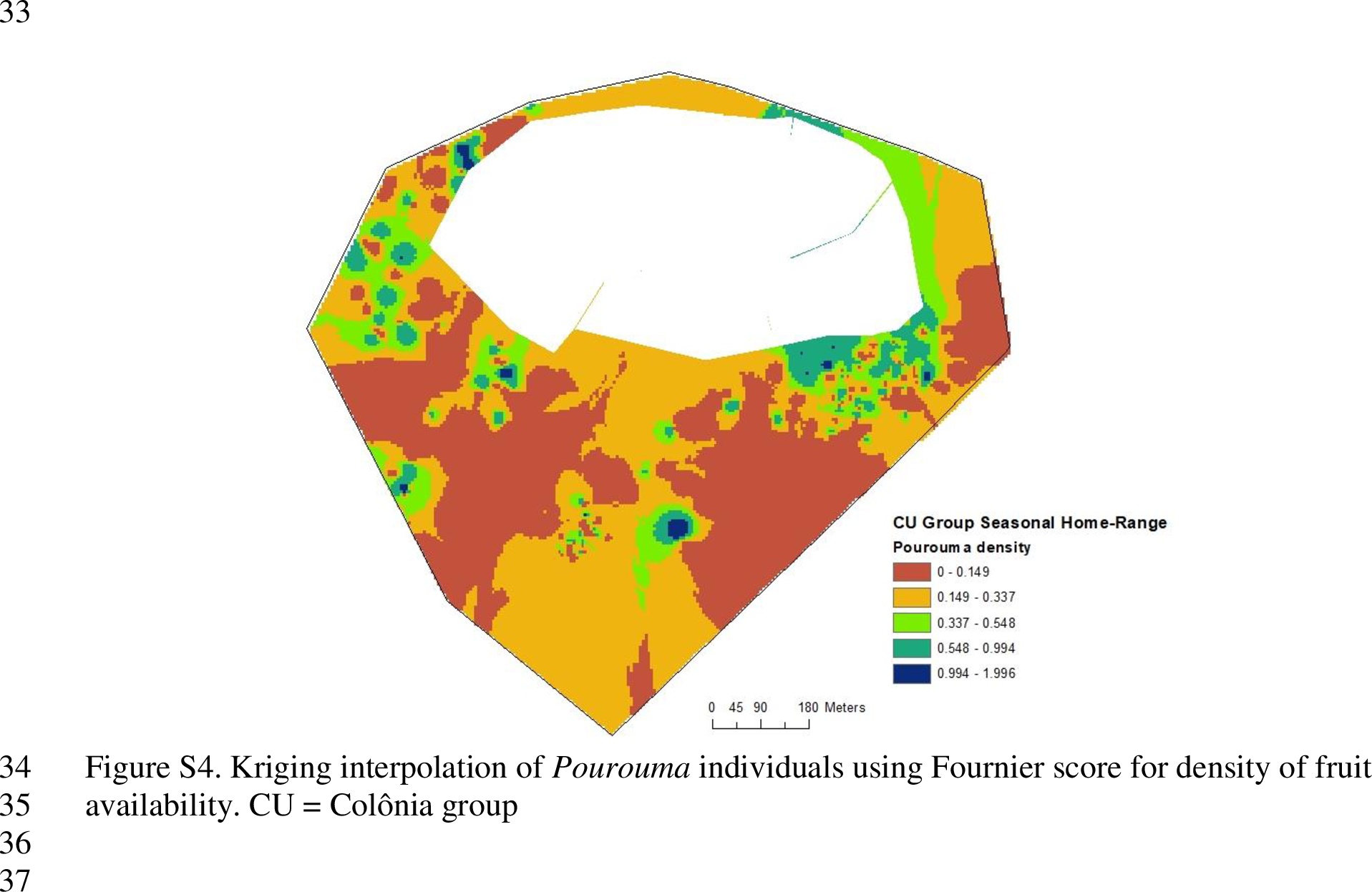

Supplement: S4 Fig — (TIF) [file pone.0244220.s004.tif]

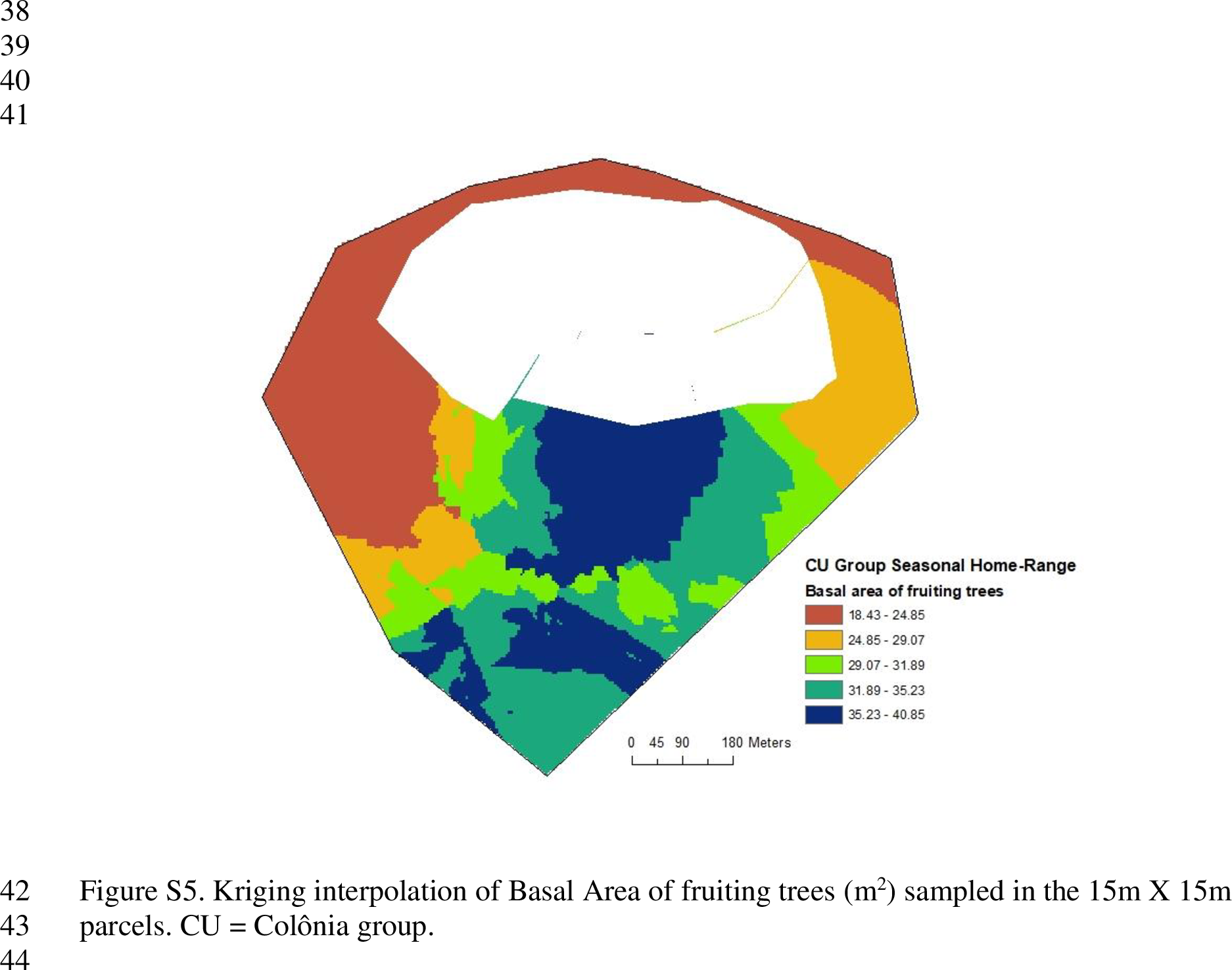

Supplement: S5 Fig — (TIF) [file pone.0244220.s005.tif]

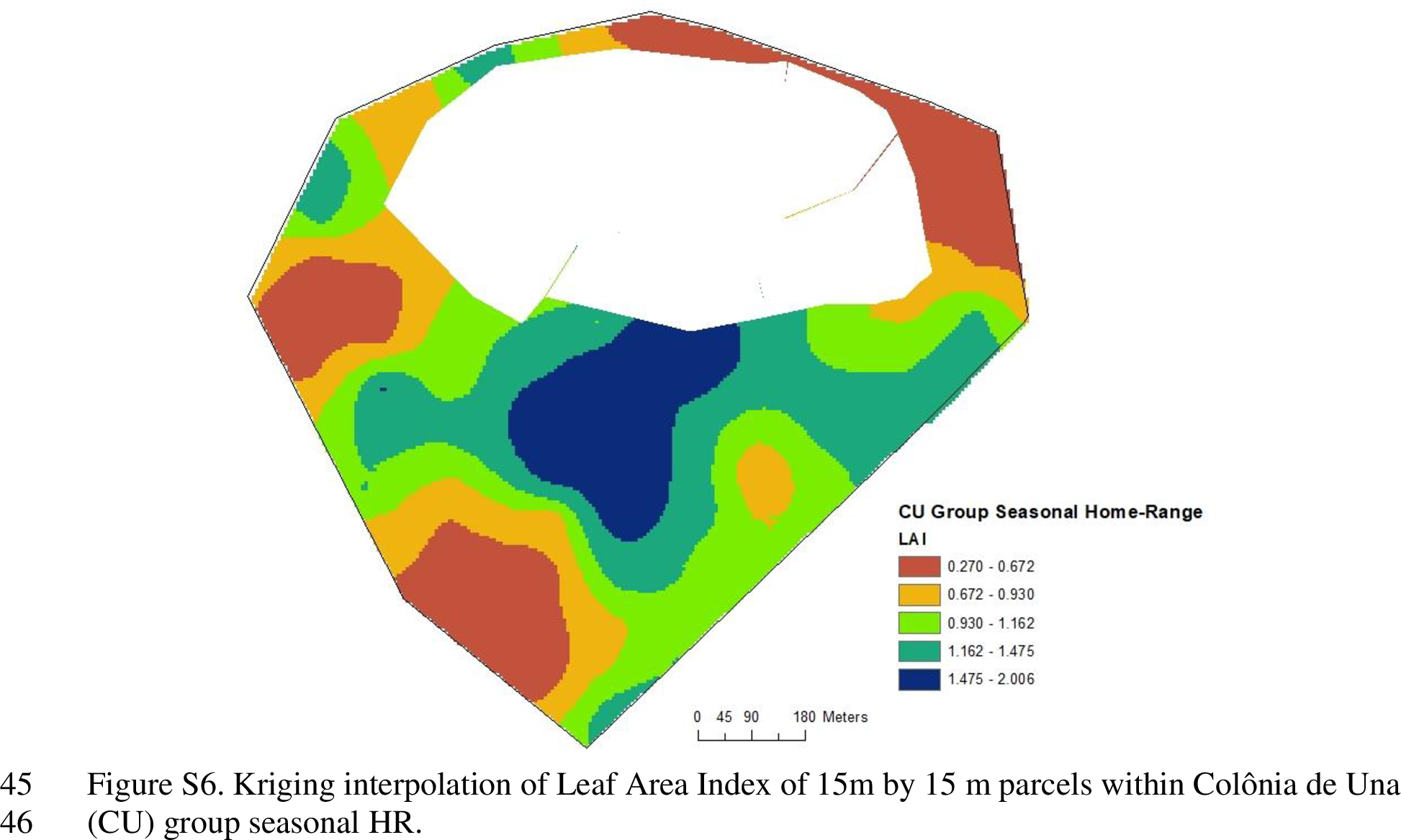

Supplement: S6 Fig — (TIF) [file pone.0244220.s006.tif]

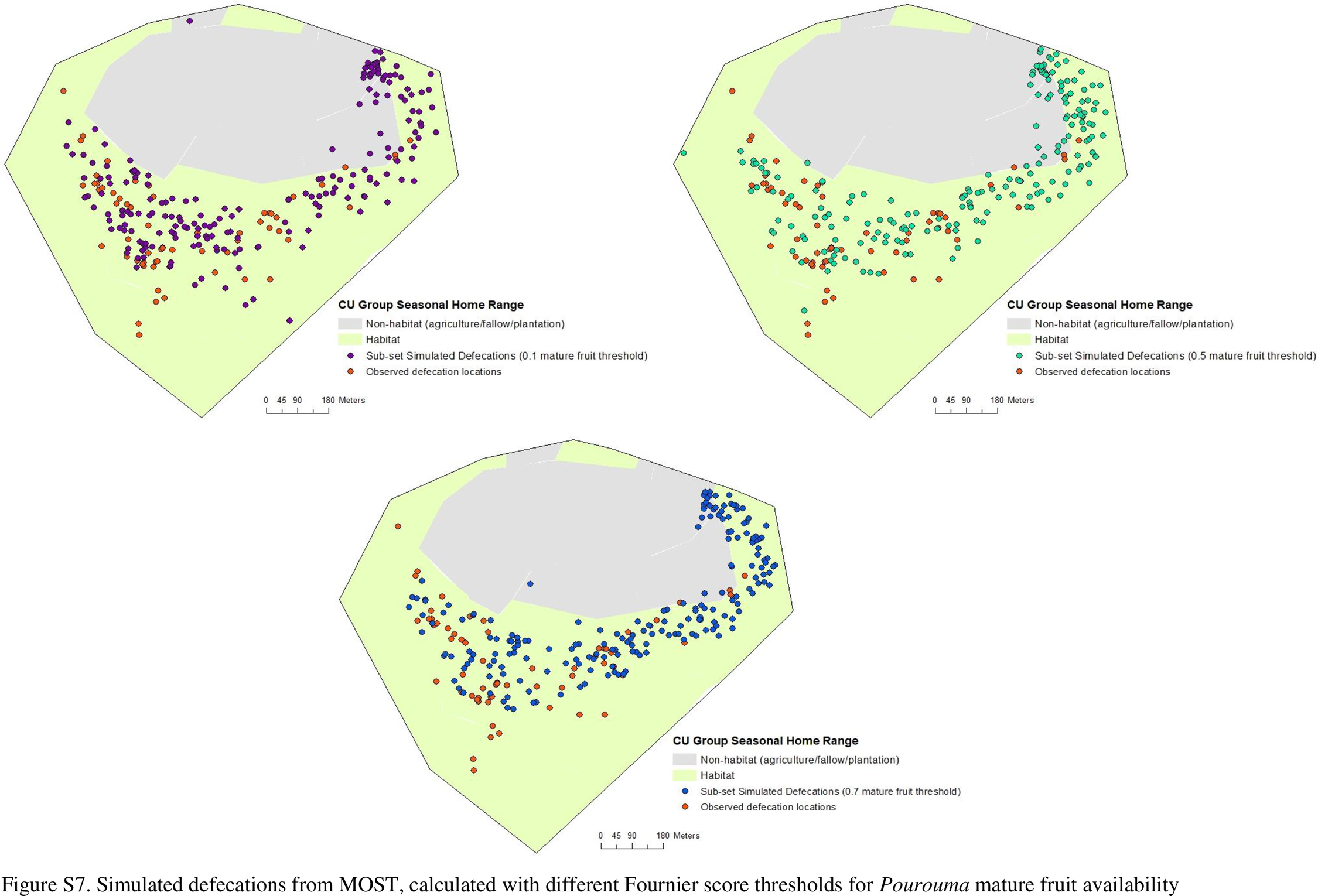

Supplement: S7 Fig — (TIF) [file pone.0244220.s007.tif]
