# Supplementary material for: Deterministic modelling of seed dispersal based on observed behaviours of an endemic primate in Brazil
Source: PLoS One. 2020 Dec 28;15(12):e0244220. doi: 10.1371/journal.pone.0244220 (PMC7769435; doi:10.1371/journal.pone.0244220)
Supplement: S1 Table — (DOC) [file pone.0244220.s008.doc]

Table S1. Frequency of visits of feeding trees in Colônia de Una, during *Pourouma* fruiting season (November – April). Some individuals were not identified and collected for future identification, indicated as “Collected” and the family, while others were only identified to genus level.

| **FID** | **Frequency of visits** | **Scntific name** |
| --- | --- | --- |
| 1 | 6 | *A. heterophyllus* |
| 2 | 4 | *Pourouma* sp. |
| 3 | 3 | *Pourouma* sp. |
| 4 | 3 | *Pourouma* sp. |
| 5 | 3 | *Henrittea succosa* |
| 6 | 2 | *Pourouma* sp. |
| 7 | 26 | *Pourouma* sp. |
| 8 | 1 | *A. heterophyllus* |
| 9 | 4 | *Pourouma* sp. |
| 10 | 4 | *Pourouma* sp. |
| 11 | 5 | *Pourouma* sp. |
| 12 | 3 | *Pourouma* sp. |
| 13 | 20 | *Pourouma* sp. |
| 14 | 4 | *Pourouma* sp. |
| 15 | 1 | *Pourouma* sp. |
| 16 | 2 | *A. heterophyllus* |
| 17 | 8 | *Pourouma* sp. |
| 18 | 2 | *Pourouma* sp. |
| 19 | 1 | *Pourouma* sp. |
| 20 | 2 | *Pourouma* sp. |
| 21 | 1 | *Pourouma* sp. |
| 22 | 5 | *Pourouma* sp. |
| 23 | 5 | *Pourouma* sp. |
| 24 | 4 | *Pourouma* sp. |
| 25 | 2 | *Pourouma* sp. |
| 26 | 1 | *Pourouma* sp. |
| 27 | 1 | *Pourouma* sp. |
| 28 | 6 | *Pourouma* sp. |
| 29 | 6 | *A. heterophyllus* |
| 30 | 13 | *A. heterophyllus* |
| 31 | 2 | *Pourouma* sp. |
| 32 | 19 | *Pourouma* sp. |
| 33 | 1 | *Pourouma* sp. |
| 34 | 3 | *Pourouma* sp. |
| 35 | 1 | *Pourouma* sp. |
| 36 | 5 | *A. heterophyllus* |
| 37 | 15 | *A. heterophyllus* |
| 38 | 2 | *Pourouma* sp. |
| 39 | 1 | *Pourouma* sp. |
| 40 | 1 | *Pourouma* sp. |
| 41 | 1 | *Pourouma* sp. |
| 42 | 2 | *Pourouma* sp. |
| 43 | 7 | *Pourouma* sp. |
| 44 | 2 | *Pourouma* sp. |
| 45 | 1 | *Pourouma* sp. |
| 46 | 4 | *Pourouma* sp. |
| 47 | 3 | *Pourouma* sp. |
| 48 | 2 | *Sejanea sp.* |
| 49 | 3 | *Pourouma* sp. |
| 50 | 1 | *Sejanea sp.* |
| 51 | 1 | Collected |
| 52 | 3 | *Pourouma* sp. |
| 53 | 1 | *Pourouma* sp. |
| 54 | 4 | Collected *Myrtaceae* |
| 55 | 1 | *Sejanea sp.* |
| 56 | 2 | *A. heterophyllus* |
| 57 | 3 | *Sejanea sp.* |
| 58 | 1 | *Sejanea sp.* |
| 59 | 1 | Collected *Myrtaceae* |
| 60 | 2 | Collected *Myrtaceae* |
| 61 | 2 | Collected *Myrtaceae* |
| 62 | 3 | *Sejanea sp.* |
| 63 | 1 | *Sorocea hilarii* |
| 64 | 4 | *Clarisia ilicifolia* |
| 65 | 1 | *Pourouma* sp. |
| 66 | 2 | Collected |
| 67 | 8 | *Protium bahianum* |
| 68 | 2 | *A. heterophyllus* |
| 69 | 3 | *Sejanea species* |
| 70 | 1 | *Sejanea species* |
| 71 | 1 | *Pourouma* sp. |
| 72 | 2 | *Clarisia ilicifolia* |
| 73 | 1 | *Syagrus botryophora* |
| 74 | 10 | *Pourouma* sp. |
| 75 | 3 | *Pourouma* sp. |
| 76 | 17 | *Pourouma* sp. |
| 77 | 1 | *Pourouma* sp. |
| 78 | 2 | *Pourouma* sp. |
| 79 | 1 | *Clarisia ilicifolia* |
| 80 | 1 | *Pourouma* sp. |
| 81 | 3 | *Pourouma* sp. |
| 82 | 6 | *Pourouma* sp. |
| 83 | 2 | *Protium heptaphyllum* |
| 84 | 1 | *Pourouma* sp. |
| 85 | 1 | *Pourouma* sp. |
| 86 | 3 | *Pourouma* sp. |
| 87 | 1 | *Pourouma* sp. |
| 88 | 2 | *Pourouma* sp. |
| 89 | 1 | *Sorocea hilarii* |
| 90 | 14 | *Pourouma* sp. |
| 91 | 12 | *Aechmea* sp. |
| 92 | 2 | *Aechmea* sp. |
| 93 | 3 | *Pourouma* sp. |
| 94 | 6 | *Aechmea* sp. |
| 95 | 1 | *Pourouma* sp. |
| 96 | 2 | *Pourouma* sp. |
| 97 | 14 | *Pourouma* sp. |
| 98 | 2 | *Protium warmigiana* |
| 99 | 1 | *Aechmea* sp. |
| 100 | 4 | *Aechmea* sp. |
| 101 | 2 | *Aechmea* sp. |
| 102 | 1 | Collected *Sapotaceae* |
| 103 | 1 | *Protium warmigiana* |
| 104 | 1 | *Cupania oblongifolia* |
| 105 | 5 | *Pourouma* sp. |
| 106 | 2 | *Inga tibaudiana* |
| 107 | 1 | *Helicostylis tomentosa* |
| 108 | 12 | *Pourouma* sp. |
| 109 | 16 | *Pourouma* sp. |
| 110 | 2 | *Aechmea blanchetiana* cf. |
| 111 | 2 | *Inga tibaudiana* |
| 112 | 1 | *Inga graziele* |
| 113 | 1 | *Pourouma* sp. |
| 114 | 4 | *Pourouma* sp. |
| 115 | 1 | *Helicostylis tomentosa* |
| 116 | 4 | *Aechmea blanchetiana* cf. |
| 117 | 1 | *Aechmea blanchetiana* cf. |
| 118 | 1 | *Inga tibaudiana* |
| 119 | 1 | *Sorocea hilarii* |
| 120 | 7 | *Aechmea* sp. |
| 121 | 1 | *Inga tibaudiana* |
| 122 | 1 | *Inga tibaudiana* |
| 123 | 2 | Collected *Sapotaceae* |
| 124 | 2 | *Inga tibaudiana* |
| 125 | 3 | *Pourouma* sp. |
| 126 | 3 | *Inga tibaudiana* |
| 127 | 1 | *A. heterophyllus* |
| 128 | 1 | *Pourouma* sp. |
| 129 | 2 | *Pourouma* sp. |
| 130 | 4 | *Pourouma* sp. |
| 131 | 1 | *Inga affinis* |
| 132 | 1 | *Helicostylis tomentosa* |
| 133 | 1 | *Inga tibaudiana* |
| 134 | 1 | *Pourouma* sp. |
| 135 | 1 | *Inga tibaudiana* |
| 136 | 2 | *Aechmea* sp. |
| 137 | 2 | *Pourouma* sp. |
| 138 | 1 | *Tapirira guianensis* |
| 139 | 1 | *Pourouma* sp. |
| 140 | 3 | *Lacmellea sp.* |
| 141 | 6 | *Inga affinis* |
| 142 | 1 | *Elaeis guianensis* |
| 143 | 1 | *Pourouma* sp. |
| 144 | 1 | *Pourouma* sp. |
| 145 | 1 | *Helicostylis tomentosa* |
| 146 | 1 | *Inga tibaudiana* |
| 147 | 4 | *Inga tibaudiana* |
| 148 | 1 | *Inga tibaudiana* |
| 149 | 1 | *Helicostylis tomentosa* |
| 150 | 1 | *Helicostylis tomentosa* |
| 151 | 1 | *Inga tibaudiana* |
| 152 | 1 | *Inga tibaudiana* |
| 153 | 1 | *Lacmellea sp.* |
| 154 | 2 | *Allophylus sericea* |
| 155 | 1 | *Inga tibaudiana* |
| 156 | 1 | *Pourouma* sp. |
| 157 | 1 | *Tapirira guianensis* |
| 158 | 2 | *Inga affinis* |
| 159 | 1 | *Inga affinis* |
| 160 | 1 | *Aechmea* sp. |
| 161 | 1 | *Inga affinis* |
| 162 | 2 | *Inga tibaudiana* |
| 163 | 1 | *Schefflera morototomi* |
| 164 | 3 | *Inga tibaudiana* |
| 165 | 1 | Collected *Chrysophyllum* |
| 166 | 1 | *Aechmea* sp. |
| 167 | 1 | *Pourouma sp.* |
| 168 | 1 | *Pourouma sp.* |
