# Supplementary material for: Deterministic modelling of seed dispersal based on observed behaviours of an endemic primate in Brazil
Source: PLoS One. 2020 Dec 28;15(12):e0244220. doi: 10.1371/journal.pone.0244220 (PMC7769435; doi:10.1371/journal.pone.0244220)
Supplement: S2 Table — (DOC) [file pone.0244220.s009.doc]

Table S2. Selection of the hidden Markov model (HMM) simulating animal movements within the home range of *Leontopithecus chrysomelas*. AIC values with combinations of the environmental variables (basal area of fruiting trees: BA, distance to sleeping site: SS, fruit availability index: FAI, distance to bromeliads: BRO, distance to fruiting trees: FT, leaf area index: LAI, distance to predator: DP, distance to other group: DG, distance to resting sites: DR) and parameters from best-fit model subsequently used in MOST.

| **Model** | **AIC** |
| --- | --- |
| BA+SS | 620.14 |
| BA+FAI | 622.76 |
| BA | 638.96 |
| DS | 649.33 |
| Null Hypothesis | 662.05 |
| BRO | 662.26 |
| FAI | 662.63 |
| DG | 664.20 |
| DR | 664.21 |
| FT | 665.52 |
| DP | 668.87 |
| FAI+SS | 806.50 |
| BA+FAI+SS | 810.80 |
| LAI | 828.43 |
