# Supplementary material for: Deterministic modelling of seed dispersal based on observed behaviours of an endemic primate in Brazil
Source: PLoS One. 2020 Dec 28;15(12):e0244220. doi: 10.1371/journal.pone.0244220 (PMC7769435; doi:10.1371/journal.pone.0244220)
Supplement: S3 Table — (DOC) [file pone.0244220.s010.doc]

Table S3. Selected model: BA+SS (Basal Area of fruiting trees + distance to Sleeping Sites)

Value of the maximum log-likelihood: -293.0703

| Gamma distribution parameters (step length) | | |
| --- | --- | --- |
|  | state 1 | state 2 |
| mean | 0.102 | 0.036 |
| sd | 0.058 | 0.026 |
| zero-mass | 0.026 | 0.154 |

| Von Mises’ distribution parameters (turning angle) | | |
| --- | --- | --- |
|  | state 1 | state 2 |
| mean | -0.018 | 0.026 |
| concentration | 0.990 | 0.415 |

| Regression coefficients for the transition probabilities | | |
| --- | --- | --- |
|  | 1🡪2 | 2 🡪 1 |
| intercept | -2.539 | -1.023e+01 |
| SS | 0.005 | 5.315e-04 |
| BA | 1.392 | 5.711e+01 |
